# Estimating the prevalence and incidence of type 2 diabetes using population level pharmacy claims data: a cross-sectional study

**DOI:** 10.1136/bmjdrc-2016-000288

**Published:** 2017-01-10

**Authors:** Sarah-Jo Sinnott, Sheena McHugh, Helen Whelton, Richard Layte, Steve Barron, Patricia M Kearney

**Affiliations:** 1 Department of Epidemiology and Public Health, University College Cork, Cork, Ireland; 2 Department of Non-communicable Disease Epidemiology, London School of Hygiene and Tropical Medicine, London, UK; 3 School of Dentistry, University of Leeds, Leeds, UK; 4 Department of Sociology, School of Social Sciences and Philosophy, Trinity College Dublin, Dublin, Ireland; 5 Economic and Social Research Institute, Dublin, Ireland

**Keywords:** Type 2, Epidemiology, Incidence, Administrative Data

## Abstract

**Objective:**

To estimate the prevalence and incidence of type 2 diabetes using a national pharmacy claims database.

**Research design and methods:**

We used data from the Health Service Executive-Primary Care Reimbursement Service database in Ireland for this cross-sectional study. Prevalent cases of type 2 diabetes were individuals using an oral hypoglycemic agent, irrespective of insulin use, in 2012. Incident cases were individuals using an oral hypoglycemic agent in 2012 who had not used one in the past. Population level estimates were calculated and stratified by age and sex.

**Results:**

In 2012, there were 114 957 prevalent cases of type 2 diabetes giving a population prevalence of 2.51% (95% CI 2.49% to 2.52%). Among adults (≥15yrs), this was 3.16% (95% CI 3.15% to 3.18%). The highest prevalence was in those aged 70+ years (12.1%). 21 574 people developed type 2 diabetes in 2012 giving an overall incidence of 0.48% (95% CI 0.48% to 0.49%). In adults, this was 0.60% (95% CI 0.60% to 0.61%). Incidence rose with age to a maximum of 2.08% (95% CI 2.02% to 2.15%) in people aged 65–69 years. Men had a higher prevalence (2.96% vs 2.04%) and incidence (0.54% vs 0.41%) of type 2 diabetes than women.

**Conclusions:**

Pharmacy claims data allow estimates of objectively defined type 2 diabetes at the population level using up-to-date data. These estimates can be generated quickly to inform health service planning or to evaluate the impact of population level interventions.

**Key messages**

- Many estimates for the burden of diabetes come from cohort studies and surveys, which are often not population based. There are very few estimates of incidence of diabetes globally.
- We used a national administrative pharmacy claims database to calculate prevalence and incidence of type 2 diabetes. This method captures all treated cases of diabetes and thus avoids problems with sampling and generalizing to whole populations that arise from cohort and survey methods.
- Understanding the true burden of disease in our society is essential for the planning of health services, which in turn help achieve optimal outcomes in terms of diabetes-related morbidity and mortality. Using pharmacy claims data is a straightforward method of achieving up to date estimates.

## Introduction

Diabetes mellitus is a leading cause of death globally, causing almost 4 million deaths in 2010.^1^ Morbidity arising from the disease is also substantial; the Global Burden of Disease study estimated a 30% increase in disability-adjusted life years for diabetes between 1990 and 2010 due to increasing prevalence of the disease and increased longevity of those living with diabetes.^2^

To reduce diabetes-related mortality and morbidity, access to appropriate healthcare services for people with diabetes is a necessity. For this to be successful, up-to-date population level estimates of disease burden are required to rationally plan and deliver the required health services.

The Institute of Public Health (IPH) estimates and forecasts for the prevalence of diabetes (combined type 1 and type 2) are often cited and are a valuable resource.^3^ However, the most recent estimates from the IPH are based on a cross-sectional survey of adults in the Survey of Lifestyle, Attitudes and Nutrition (SLAN) which dates back to 2007.^3^ ^4^

Other estimates of diabetes prevalence come from The Irish Longitudinal Study of Ageing (TILDA), which is limited to those over 50 years,^5^ ^6^ the Mitchelstown cohort which was limited to adults aged 50–69 years in one rural area in the South of Ireland^7^ and the Central Statistics Office (CSO) Quarterly National Household Survey (QNHS) from 2010.^8^ These estimates are based on a variety of self-reported doctor diagnosis; self-reported diabetes medication usage; or a combination of self-report and HbA1c data; and all are limited to adult populations. Furthermore, there is a scarcity of data available on the incidence of diabetes in Ireland.^9^

In this study, we used national pharmacy claims data to estimate the prevalence and incidence of type 2 diabetes. These data provide an objective measure of treated diabetes in the total population to complement existing sample-based estimates.

## Methods

### Health system

In Ireland, access to and reimbursement for diabetes medicines occur via two publicly funded community drug schemes. The first is the General Medical Services (GMS) scheme; the main public health insurance program providing primary and secondary healthcare free at the point of access to ∼40% of the Irish population on a means-tested basis.^10^ Medicines are included under this scheme but are subject to a copayment (€2.50 currently). The second drug scheme is the long-term illness (LTI) scheme. The LTI provides free access to condition-related medicines for individuals diagnosed with any of 16 chronic illnesses including diabetes. LTI coverage is independent of income.

### Data

Pharmacists dispensing medicines to all patients (adults and children) on the GMS and the LTI scheme are reimbursed by the government via the Health Service Executive-Primary Care Reimbursement Service (HSE-PCRS). We used dispensing data from the HSE-PCRS database from July 2011 to December 2012. Data were available for the drug dispensed (classified by WHO Anatomical Therapeutic Chemical (WHO ATC) code), date dispensed, quantity and strength, in addition to patient age and sex.

Population denominator data for the year 2012 were population estimates derived by the CSO based on the 2011 census.^11^

### Definitions

Type 2 diabetes was classified as using any strength or quantity of an oral hypoglycemic agent (WHO ATC A10B), irrespective of age or insulin use. The different agents included in this study, stratified by age and sex, are given in table 1.

**Table 1:** Types of medicines used in 2012

Medicine group	Biguanides	Sulfonylureas	Thiazolidinediones	DPP-4	Other	Total
	A10BA	A10BB	A10BG	A10BH	A10BC, A10BF, A10BX	n (%)
WHO ATC code	n (%)	n (%)	n (%)	n (%)	n (%)	
Total	907 125 (51.9)	527, 886 (30.2)	25 658 (1.5)	126 345 (7.2)	60 069 (3.4)	1 747 755 (100.0)
<15 years	1547 (50.0)	914 (29.5)	50 (1.6)	142 (4.6)	139 (4.5)	3096 (0.18)
15–24 years	2959 (69.7)	675 (15.9)	57 (1.3)	178 (4.2)	212(5.0)	4247 (0.24)
25–34 years	12 537 (67.6)	3020 (16.3)	247 (1.3)	734(4.0)	1088 (5.9)	18 554 (1.06)
35–44	48 558 (59.3)	17 542 (21.4)	1243 (1.5)	4414 (5.4)	4787 (5.8)	81 960 (4.7)
45–54 years	125 732 (54.0)	57 833 (24.8)	3553 (1.5)	14 760 (6.3)	14 168 (6.1)	232 955 (13.3)
55–64 years	236 515 (52.6)	122 554 (27.3)	7493 (1.7)	31 253 (6.9)	20 675 (4.6)	449 432 (25.7)
65–69 years	140 569 (52.0)	79 897 (29.6)	4372 (1.6)	20 725 (7.7)	7936 (2.9)	270 106 (15.5)
70+ years	333 853 (49.3)	242 256 (35.8)	8435 (1.3)	53 435 (7.9)	10 600 (1.6)	677 401 (38.8)
Women	368 707 (53.2)	204 465 (29.5)	9450 (1.4)	49 794 (7.2)	25 412 (3.7)	692 565 (39.6)
Men	536 766 (51.0)	322 465 (30.7)	16 073 (1.5)	76 275 (7.3)	34 545 (3.3)	1 051 910 (60.2)

Numbers are numbers of prescriptions in 2012.

Other includes sulfonamides, α glucosidase inhibitors and ‘other’ agents as defined by WHO ATC dictionary.

DPP-4, dipeptidyl peptidase-4 inhibitors.

### Calculation of incidence and prevalence

To estimate the prevalence of type 2 diabetes, we used dispensing data for 2012. We counted the number of people in the database who met our definition of type 2 diabetes and used this as the numerator. The total population count published by the CSO was the denominator.^11^

To establish the annual incidence for type 2 diabetes in 2012, we used data from July 2011 to December 2012. An individual's first occurrence in 2012 meeting the definition of type 2 diabetes was referred to as the index date. A 6 month look-back period was used to rule out prior use before the index date. If no prior use of an oral hypoglycemic agent occurred in the look-back period, then the individual was an incident user of oral hypoglycemic medicines and thus an incident case. This count was used as the numerator, while the denominator was the total population count published by the CSO minus the number of prevalent of cases.^11^

We carried out subgroup analyses by age group (<15 years, ≥15 years, 15–24 years, 25–34 years, 35–44 years, 45–54 years, 55–64 years, 65–69 years and >70+ years) and sex.

## Results

In 2012, 1 655 013 people accessed a prescription on the GMS scheme and were available in our data set. The mean age was 42.9 years (SD 25.9), and the population was 54.4% women. On the LTI scheme, 68 996 people accessed at least one prescription in 2012. The mean age was 48.4 years (SD 25.2), and the population was 38% women.

### Type 2 diabetes mellitus

**Figure 1:**
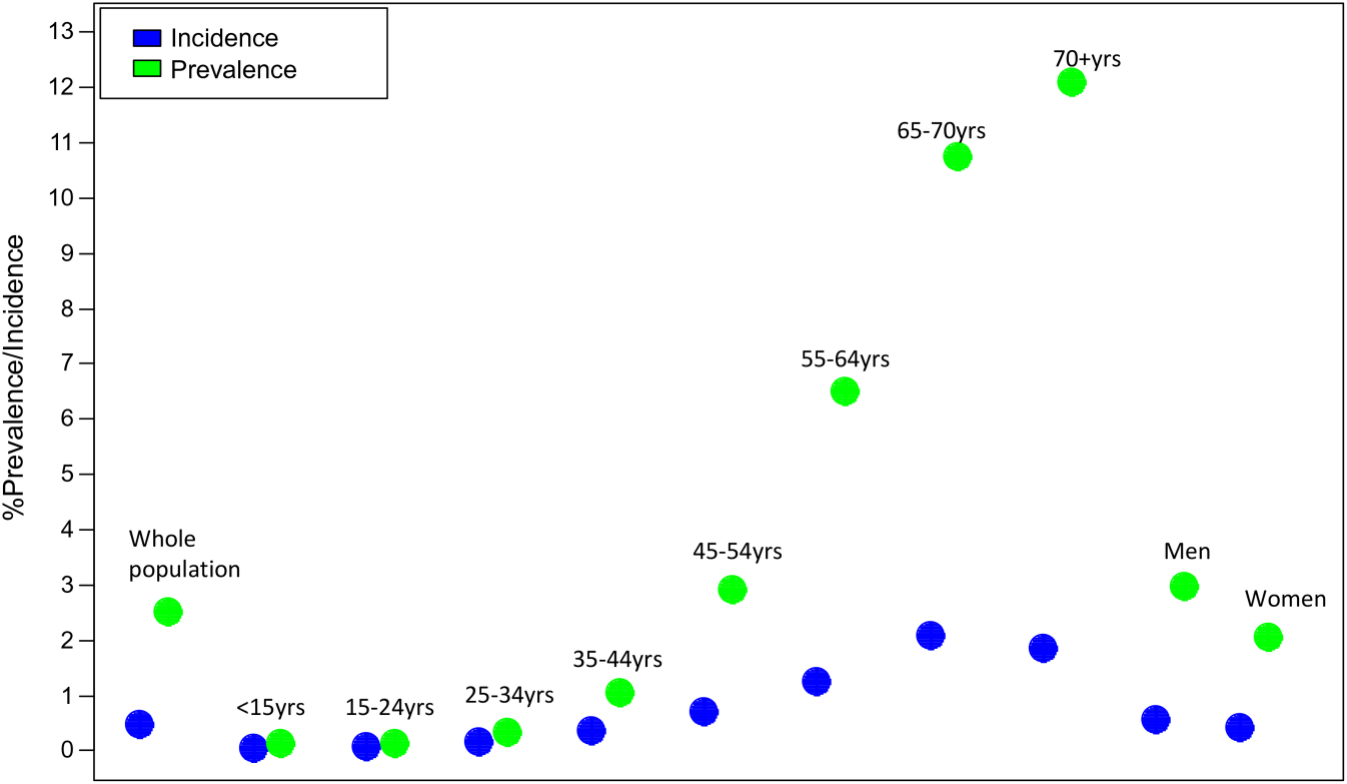
Prevalence and incidence of type 2 diabetes.

In 2012, 114 957 people were classified as prevalent type 2 diabetes cases, leading to a prevalence of 2.51% (95% CI 2.49% to 2.52%) in the total population. After excluding those aged <15 years, an adult population prevalence of 3.16% (95% CI 3.15% to 3.18%) was obtained (table 2). Figure 1 demonstrates how the prevalence increased with age; 55–64 years (6.50%), 65–69 years (10.75%) and 70+ years (12.10%). Men had a higher prevalence of type 2 diabetes than women at 2.96% (95% CI 2.94 to 2.98) vs 2.04% (95% CI 2.02% to 2.06%) (χ^2^ test for homogeneity p<0.0001).

**Table 2:** Prevalence and incidence of type 2 diabetes

	GMS	LTI	Total	Population (CSO)	Estimate (%)	95% CI
Prevalence estimates						
Total population	81 177	33 780	114 957	4 585 000	2.51	2.49 to 2.52
Total population ≥15 years	80 618	32 987	113 605	3 590 600	3.16	3.15 to 3.18
<15 years	721	1198	1919	994 800	0.19	0.18 to 0.20
15–24 years	594	128	722	553 500	0.13	0.12 to 0.14
25–34 years	1702	765	2467	733 500	0.34	0.32 to 0.35
35–44 years	4309	3081	7390	700 000	1.06	1.03 to 1.08
45–54 years	8964	8088	17 052	586 300	2.91	2.87 to 2.95
55–64 years	16 466	13 938	30 404	468 000	6.50	6.43 to 6.57
65–69 years	12 396	7120	19 516	181 500	10.75	10.61 to 10.90
70+ years	41 098	3397	44 495	367 800	12.10	11.99 to 12.20
Women	36 968	10 231	47 199	2 315 800	2.04	2.02 to 2.06
Men	43 707	23 510	67 217	2 269 600	2.96	2.94 to 2.98
Incidence estimates						
Total population	15 788	5786	21 574	4 470 043	0.48	0.48 to 0.49
Total population ≥15 years	15 353	5679	21 032	3 476 995	0.60	0.60 to 0.61
<15 years	213	234	447	992 881	0.05	0.04 to 0.05
15–24 years	405	55	460	552 678	0.08	0.08 to 0.09
25–34 years	936	293	1229	731 033	0.17	0.16 to 0.18
35–44 years	1584	819	2403	692 610	0.35	0.33 to 0.36
45–54 years	2459	1523	3982	569 248	0.70	0.68 to 0.72
55–64 years	3485	2026	5511	437 596	1.26	1.23 to 1.29
65–69 years	2406	971	3377	161 984	2.08	2.02 to 2.15
70+ years	5585	413	5998	323 305	1.86	1.81 to 1.9
Women	7242	1959	9201	2 268 601	0.41	0.40 to 0.41
Men	8165	3815	11 980	2 202 383	0.54	0.53 to 0.55

In the same year, 21 574 people developed type 2 diabetes giving an incidence of 0.48% (95% CI 0.48% to 0.49%). This was estimated at 0.60% (95% CI 0.60% to 0.61%) in the population aged ≥15 years. The incidence of type 2 diabetes increased with age, reaching its highest level of 2.08% (95% CI 2.02 to 2.15) in people aged 65–69 years. Men had a higher incidence of type 2 diabetes (0.54%) than women (0.41%) (table 2 and figure 1).

## Discussion

This cross-sectional study estimated the prevalence and incidence of type 2 diabetes using population level data from a national pharmacy claims database. The overall prevalence in the adult population was 3.16%. The incidence of type 2 diabetes was 6 cases per 1000 adult people in 2012.

Existing prevalence estimates pertaining to the general adult population range from 3% in the Quarterly National Household survey to 3.5% in those aged ≥18 years using SLAN survey data.^4^ ^8^ Our estimate of 3.16% is thus comparable to previous figures. In addition, our age stratified estimates for people aged ≥50 years are similar to those based on TILDA using self-report of doctor diagnosis and HbA1c measures.^5^ ^6^ However, our estimates may underestimate the true burden of diabetes given that we have excluded type 1 diabetes and we also could not account for lifestyle-treated diabetes or undiagnosed diabetes. Despite this, the true prevalence rate of diabetes in Ireland is likely lower than that in the USA which was recently estimated at 8.3% in the adult population.^12^ In England, diagnosed diabetes in the population aged ≥16 years is estimated at 5.6% from Health Survey for England data.^13^

The only other estimate of incidence of type 2 diabetes in Ireland is 2 cases per 1000, in contrast to the 6 cases per 1000 we found in this study.^9^ An American study using the National Health Interview Survey found an incidence rate of 7.1/1,000 people aged ≥20 years in 2012, indicating that Irish incidence rates are below those in North America.^12^ A recent Danish study calculated incidence rates for every year of age.^14^ While it is difficult to compare single age estimates with estimates for age categories, our estimates appear comparable to those in the Danish study, albeit somewhat higher in the older age groups.^14^ Making international comparisons is helpful to aid in understanding the plausibility of our estimates, however differences do exist between populations for demographic and methodological reasons.^15^

The study is limited by lack of information on undiagnosed diabetes and lifestyle-treated diabetes. Other data sources, for example the Mitchelstown Cohort and SLAN survey, provide information on undiagnosed diabetes, which can be used in tandem with our results.^4^ ^7^ Unpublished data from the Mitchelstown Cohort study of over 2000 adults aged 50–69 years reveal that ∼7% of those with self-reported diabetes are treated with diet only.^16^ Although these data are not nationally representative, they provide some context on the magnitude of underestimation. Furthermore, we relied on diagnosed individuals adhering to their treatment regimens, their dispensed medications thus appearing in the pharmacy claims database. We did not anticipate non-adherence to be a major problem given that medicines are free on the LTI scheme and subject to a small copayment on the GMS scheme (€0.50 per item in 2012).^17^

The study is strengthened by the objective and reliable nature of the data.^18^ Additionally, because diabetes medicines are generally provided only through the GMS and LTI drug schemes, data on those with diagnosed and treated diabetes should be nationally complete in this database offering population level data for all ages, including children, in contrast to previous surveys and cohort studies which are limited to adults. Unfortunately, the database does not have access to diagnosis codes, thus we made the assumption that all oral hypoglycemic agents were being used to treat diabetes. A notable exception is the use of metformin for polycystic ovarian syndrome (PCOS). However, as PCOS effects only a small proportion of women of reproductive age, ∼8%, and only some of these will be treated, any effect on our estimates is likely to be small. Further, many women with PCOS will have diabetes, and we will have intended to include them in our estimates.^19^ Metformin is also used in pre-diabetes, which due to lack of diagnosis codes, we have not been able to separate from our estimates. We know from our prior research that the prevalence of pre-diabetes in those aged ≥45 years is 20%.^20^ From the most recent audit of diabetes management in General Practice, we know that ∼90% of those with pre-diabetes are treated with dietary intervention (unpublished).^21^ Thus, any bias contributed to our results from including those with metformin-treated pre-diabetes is likely to be inconsequential.

Our study has demonstrated the utility of routinely collected administrative claims data in calculating measures of disease burden, including incidence. The method is straightforward, and while we used data from 2012 for this study, more current data would allow estimating disease burden for the most recently completed calendar year along with establishing longitudinal trends. This approach affords advantages in real-time monitoring of disease burden and thus presents a key resource for evaluating public health interventions to reduce the prevalence and incidence of type 2 diabetes, thus informing health policy and health service planning. To address acknowledged weaknesses in using these data, estimates for prevalence and incidence should be considered in combination with cross-sectional and cohort study results to account for undiagnosed and lifestyle-treated cases.

## References

[1] Roglic G, Unwin N Mortality attributable to diabetes: estimates for the year 2010. Diabetes Res Clin Pract 2010;87:15–19. 10.1016/j.diabres.2009.10.006 19914728

[2] Murray CJ, Vos T, Lozano R, et al Disability-adjusted life years (DALYs) for 291 diseases and injuries in 21 regions, 1990–2010: a systematic analysis for the Global Burden of Disease Study 2010. Lancet 2013;380:2197–223. 10.1016/S0140-6736(12)61689-4 23245608

[3] Balanda KP, Jordan A and McArdle E (2006) Making diabetes count. A systematic approach to estimating population prevalence on the island of Ireland in 2005. Dublin/Belfast: Institute of Public Health in Ireland.

[4] Balanda KP, Buckley CM, Barron SJ, et al Prevalence of diabetes in the Republic of Ireland: results from The National health survey (SLAN) 2007. PLoS One 2013;8 10.1371/journal.pone.0078406 PMC3797781 24147134

[5] Tracey M, McHugh S, Buckley C, et al The prevalence of Type 2 diabetes and related complications in a nationally representative sample of adults aged 50 and over in the Republic of Ireland. Diabet Med 2016;33:441–5. 10.1111/dme.12845 26112979

[6] Leahy S, O'Halloran A, O'Leary N, et al Prevalence and correlates of diagnosed and undiagnosed type 2 diabetes mellitus and pre-diabetes in older adults: findings from the Irish Longitudinal Study on Ageing (TILDA). Diabetes Res Clin Pract 2015;110: 241–9. 10.1016/j.diabres.2015.10.015 26520567

[7] Connor JM, Millar SR, Buckley CM, Kearney PM and Perry IJ, et al 2013. The prevalence and determinants of undiagnosed and diagnosed type 2 diabetes in middle-aged irish adults PloS one, 8(11), p.e80504. 10.1371/journal.pone.0080504 PMC3840064 24282548

[8] Central Statistics Office. Health status and health service utilisation. Quarterly National Household Survey. Quarter 3, 2010 2010 http://www.cso.ie/en/media/csoie/releasespublications/documents/labourmarket/2010/healthstatusq32010.pdf (accessed 23rd December 2016).

[9] Tracey, Marsha L., *et al*. Epidemiology of diabetes and complications among adults in the Republic of Ireland 1998-2015: a systematic review and meta-analysis. *BMC public health* 16.1 (2016):1. 10.1186/s12889-016-2818-2 PMC4748605 26861703

[10] Health Service Executive. Primary care reimbursement service—statistical analysis of claims and payments 2012 http://www.hse.ie/eng/staff/PCRS/PCRS_Publications/PCRSannreport12.pdf (accessed 18 Jan 2016).

[11] Central Statistics Office. Population and migration estimates 2012 http://www.cso.ie/en/media/csoie/releasespublications/documents/population/2012/popmig_2012.pdf (accessed 23rd December 2016).

[12] Geiss LS, Wang J, Cheng YJ, et al Prevalence and incidence trends for diagnosed diabetes among adults aged 20 to 79 years, United States, 1980–2012. JAMA 2014;312:1218–26. 10.1001/jama.2014.11494 25247518

[13] Mainous AG, Tanner RJ, Baker R, et al Prevalence of prediabetes in England from 2003 to 2011: population-based, cross-sectional study. BMJ Open 2014;4:e005002 10.1136/bmjopen-2014-005002 PMC4054625 24913327

[14] Carstensen B, Kristensen JK, Ottosen P, et al The Danish National Diabetes Register: trends in incidence, prevalence and mortality. Diabetologia 2008;51:2187–96. 10.1007/s00125-008-1156-z 18815769

[15] Fazeli Farsani S, van der Aa MP, van der Vorst MM, et al Global trends in the incidence and prevalence of type 2 diabetes in children and adolescents: a systematic review and evaluation of methodological approaches. Diabetologia 2013;56:1471–88. 10.1007/s00125-013-2915-z 23677041

[16] Kearney PM, Harrington JM, Mc Carthy VJ, et al Cohort profile: the Cork and Kerry diabetes and heart disease study. Int J Epidemiol 2013;42:1253–62. 10.1093/ije/dys131 22984148

[17] Sinnott SJ, Normand C, Byrne S, et al Copayments for prescription medicines on a public health insurance scheme in Ireland. Pharmacoepidemiol Drug Saf 2016;25:695–704. 10.1002/pds.3917 26696242

[18] Grimes T, Fitzsimons M, Galvin M, et al Relative accuracy and availability of an Irish National Database of dispensed medication as a source of medication history information: observational study and retrospective record analysis. J Clin Pharm Ther 2013;38:219–24. 10.1111/jcpt.12036 23350784

[19] Sirmans SM, Pate KA Epidemiology, diagnosis, and management of polycystic ovary syndrome. Clin Epidemiol 2014;6:1. 10.2147/CLEP.S37559 PMC3872139 24379699

[20] Buckley CM, Madden J, Balanda K, et al Pre-diabetes in adults 45 years and over in Ireland: the Survey of Lifestyle, Attitudes and Nutrition in Ireland 2007. Diabet Med 2013;30:1198–203. 10.1111/dme.12226 23659572

[21] Murphy K, Mc Hugh S, Moran J Diabetes in general practice audit report; Jun 2009–May 2010 2011.

